# Cleanliness by Law

**Published:** 1921-01-08

**Authors:** 


### Cleanliness by Law.

A London coroner did good service a few days ago at an inquest on a woman who died as the result of self-neglect by calling attention to the necessity for a wider application of compulsory cleanliness. The Coroner's remarks led the jury to add to their verdict a rider in which they expressed the view that the adoptive Act, known as the Cleansing of the People Act, should be made compulsory, and that the police should take into charge people found in public places visibly suffering from dirt and should cleanse them. This Act is little known, and we are glad of the opportunity of bringing it to the notice of local authorities. But it will not effect the purpose for which it is designed until it is strengthened and made compulsory. Dirt is the handmaid of disease, and dirt must be destroyed wherever it is to be found. The liberty of the subject is quite properly interfered with in the case of infectious disease. The subject has still less right to complain if the community insists in seeing that he is reasonably clean.

